# ^31^P ParaCEST: ^31^P MRI-CEST Imaging Based on the Formation of a Ternary Adduct between Inorganic Phosphate and Eu-DO3A

**DOI:** 10.1021/acs.inorgchem.2c03329

**Published:** 2022-11-29

**Authors:** Giulia Vassallo, Francesca Garello, Silvio Aime, Enzo Terreno, Daniela Delli Castelli

**Affiliations:** † Department of Molecular Biotechnology and Health Science, University of Turin, Via Nizza 52, 10126Turin, Italy; ‡ IRCCS SDN SynLab, Via E. Gianturco 113, 80143Napoli, Italy

## Abstract

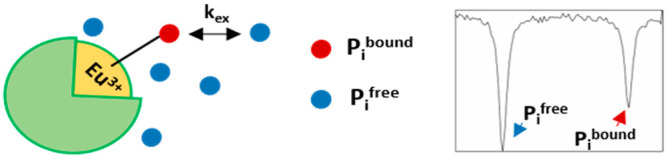

Development of the field of magnetic resonance imaging (MRI) chemical exchange saturation transfer (CEST) contrast agents is hampered by the limited sensitivity of the technique. In water, the high proton concentration allows for an enormous amplification of the exchanging proton pool. However, the ^1^H CEST in water implies that the number of nuclear spins of the CEST-generating species has to be in the millimolar range. The use of nuclei other than a proton allows exploitation of signals different from that of water, thus lowering the concentration of the exchanging pool as the source of the CEST effect. In this work, we report on the detection of a ^31^P signal from endogenous inorganic phosphate (P_i_^free^) as the source of CEST contrast by promoting its exchange with the P_i_ bound to the exogenous complex 1,4,7,10-tetraazacyclododecane-1,4,7-triacetic acid (P_i_^bound^). The herein-reported results demonstrate that this approach can improve the detectability threshold by 3 orders of magnitude with respect to the conventional ^1^H CEST detection (considered per single proton). This achievement reflects the decrease of the bulk concentration of the detected signal from 111.2 M (water) to 10 mM (P_i_). This method paves the way to a number of biological studies and clinically translatable applications, herein addressed with a proof-of-concept in the field of cellular imaging.

## Abstract 2

This work illustrates the possibility to perform ^31^P-based chemical exchange saturation transfer magnetic resonance imaging detection by exploiting the interaction between a paramagnetic complex and the biologically relevant inorganic phosphate (P_i_). A detectability enhancement of 3 orders of magnitude in the concentration of the paramagnetic probe was observed. A proof-of-concept of the potential of this approach in cellular imaging is provided.

The magnetic resonance imaging chemical exchange saturation transfer (MRI-CEST) technique was proposed (and generally intended) to generate a frequency-encoded contrast on the “bulk” water resonance through the exploitation of molecules endowed with exchangeable protons.^1−4^ This indirect contrast allows for an amplification of the proton MRI detection threshold of these molecules by exploiting their effect on the much more intense signal of the bulk water. The experiment consists of saturating the NMR signal of the protons in chemical exchange with water and then measuring the saturation transfer (ST) on the NMR signal of the bulk solvent. The ST efficiency depends on several parameters (among which are the exchange rate of the mobile protons, the intensity, shape, and duration of the saturation field, the magnetic field strength, etc.), and it is proportional to the fraction of saturated exchanging nuclei belonging to the contrast agent (*N*°_CA_) over the arrival, detected, spins (*N*°_Bulk_):
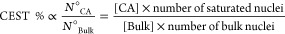
where [CA] is the concentration of the CEST contrast agent and [Bulk] is the concentration of the MRI-detected pool. Considering ^1^H MRI-CEST, the arrival pool is the bulk water (*n* = 2), whose concentration is approximately 55.6 M. Such a high value severely limits the efficiency of proton CEST detection, whose lower bound, as considered per single proton resonance, is in the millimolar range. It goes without saying that a reduction of the [Bulk] term yields to proportionally improve the detection threshold, thus allowing the indirect visualization of nuclei present in concentrations much lower than millimolar. Recently, Bar-Shir and co-workers applied this concept,^5,6^ showing a 900-fold signal amplification in the ^19^F MRI-CEST detection of an inhalable fluorinated anesthetic. The required shift of the resonance to be saturated was obtained by promoting a host–guest supramolecular interaction with a macrocyclic ligand. This approach led to an increase in the CEST detectability (per nucleus) of 3 orders of magnitude, enabling a micromolar detection of the saturated pool, due to a reduction in the concentration of the *bulk* site. An analogous approach was at the basis of the development of ^129^Xe-based hyperCEST.^7,8^ Inspired by these works, we deemed it of interest to explore a route for the detection of endogenous ^31^P resonances. ^31^P ST experiments were reported as early as the 1980s and were used to assess the exchange rate between inorganic phosphate (P_i_) and γ-adenosine triphosphate (γ-ATP) in kidneys or phosphocreatine and γ-ATP in the heart; despite this, ^31^P as the source of MRI-CEST contrast agents had never been proposed.^9,10^ Indeed, ^31^P has sufficient NMR receptivity (ca. 7% compared to ^1^H) and it is present in different endogenous molecules at sufficiently high concentrations to directly act as the reporter of the CEST effect. Among the ^31^P-containing endogenous molecules, P_i_ appears to be an excellent candidate because it displays a quite constant concentration in different physiological fluids (about 1–3 mM in the extracellular medium and 10 mM in the intracellular compartment).^11,12^ To create the conditions to detect ^31^P MRI-CEST on P_i_ (i.e., to have two exchangeable P_i_ sites with sufficiently different chemical shift values), we exploited the interaction between P_i_ and a coordinatively unsaturated paramagnetic lanthanide complex, here represented by 1,4,7,10-tetraazacyclododecane-1,4,7-triacetic acid (Eu-DO3A; Scheme 1).^13−16^ The large shift induced on the coordinated phosphate moiety (P_i_^bound^) allows one to match the basic conditions for CEST detection (Δω > *k*_ex_).

**Scheme 1:**
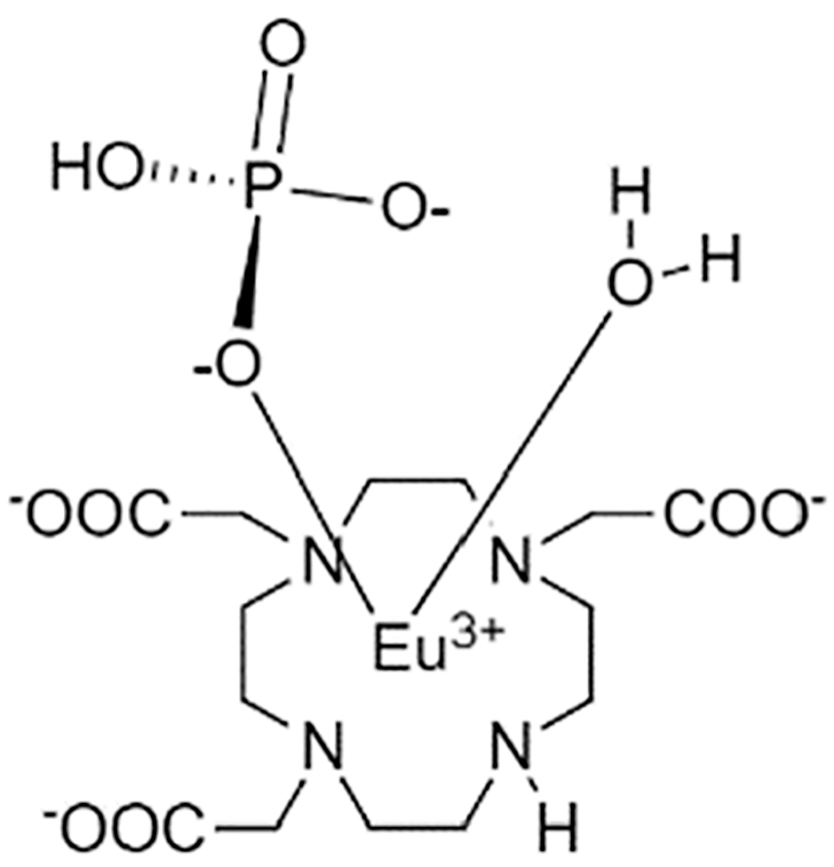
Schematic Representation of the Ternary Adduct between Eu-DO3A and P_i_ (in the HPO_4_^2–^ Form)

The ^31^P NMR spectrum (14 T, 295 K) of an aqueous solution containing Eu-DO3A (40 μM) and P_i_ (10 mM) at pH 7.0 showed only the P_i_^free^ signal because the P_i_^bound^ signal could not be observed due to its very low concentration (and likely for the signal broadening induced by coordination to the paramagnetic center). However, when a ^31^P Z-spectrum was acquired for the same solution at 295 or 310 K, sharp CEST peaks at −134 and −120 ppm, respectively, from P_i_^free^ were observed, which highlighted the occurrence of a large ST effect (ca. 70% at 295 K and 49% at 310 K) consequent to saturation of the P_i_^bound^ pool (Figure 1). Measuring the CEST effect as a function of the intensity of the saturation pulse (the so-called ω plot),^17^ the exchange rates of P_i_^bound^ were calculated to be 2.10 and 3.03 kHz at 295 and 310 K, respectively, values sufficiently smaller than Δω (ca. 6 kHz), thus confirming a match with the condition required to detect CEST contrast.

**Figure 1:**
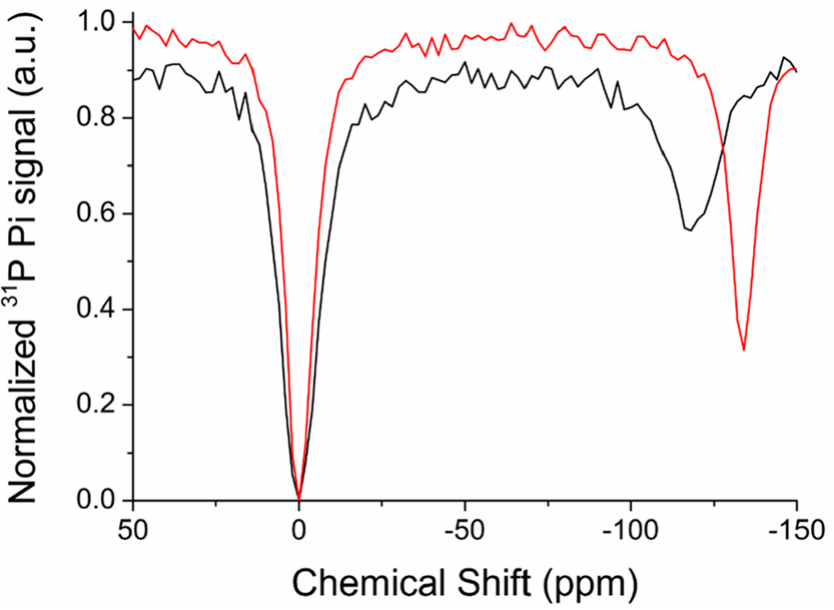
^31^P Z-spectrum at 14 T of a solution containing 40 μM Eu-DO3A and 10 mM P_i_ (pH 7.0). Acquisition temperatures: 295 K, red line; 310 K, black line. The peak at 0 ppm corresponds to the saturation of P_i_^free^, whereas the peak at −134 or −120 ppm refers to the saturation of P_i_ bound to Eu-DO3A. Acquisition parameter: 150 points; 16 scans each; *B*_1_ = 22 μT.

Next, the ^31^P ST % contrast was measured as a function of the concentration of Eu-DO3A (Figure 2A) to assess the minimum amount of paramagnetic probe necessary to achieve the ST detection limit, placed at 5%. The obtained value, around 2.5 μM, was about 3 orders of magnitude lower than the amount required for ^1^H CEST detection for conventional paraCEST agents.^18−20^

**Figure 2:**
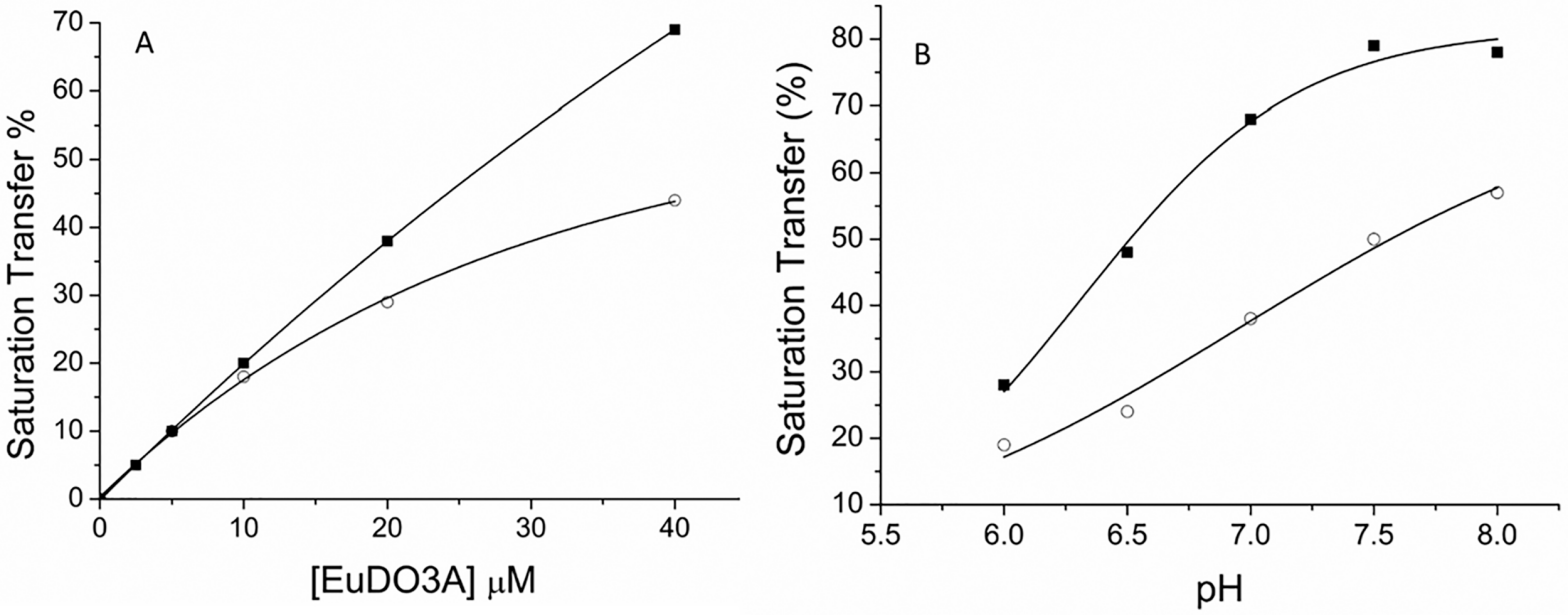
(a) Plot of ST as a function of the concentration of Eu-DO3A in a solution of 10 mM P_i_ at pH 7.0 (black squares, 295 K; empty circles, 310 K). (b) pH dependence of the ^31^P ST effect for a solution containing a 40 μM Eu-DO3A complex in a solution of 10 mM P_i_, measured upon saturation of the frequency for the individual pH readout (black squares, 295 K; empty circles, 310 K).

The pH dependence of the ^31^P ST effect was measured in the pH 6–8 interval. As well-documented by others,^21−23^ the chemical shift difference between P_i_^free^ and P_i_^bound^ showed a clear pH dependence (Figure S1). The pH dependence of the ST contrast at 295 K (Figure 2B), measured upon saturation of the resonance frequency at the Δω values reported in Figure S1, showed a steep linear increase between pH 6 and 7, followed by deflection and stabilization of the effect between pH 7.5 and 8.

The observed behavior can be interpreted by considering the pH-dependent speciation of P_i_. The p*K*_a_ for the acid/base equilibrium for the H_2_PO_4_^–^/HPO_4_^2–^ conjugated pair (H_2_PO_4_^–^ ⇌ HPO_4_^2–^ + H^+^) is 7.2. This means that in the examined pH range the molar fraction of HPO_4_^2–^ rises from 10% to 90%. Because binding of the Eu-DO3A complex to HPO_4_^2–^ is likely stronger than that to H_2_PO_4_^–^, the concentration of P_i_^bound^ increases upon moving from pH 6 to 8, thus enhancing the ST efficiency. However, beyond pH 7, ST % reached a plateau. Likely, this finding suggests that the CEST maximum is the result of a balance between the highest concentration of HPO_4_^2–^ and the increase in the exchange rate of P_i_^bound^, which may reduce the ST efficiency. The same consideration can be done for the pH dependence at 310 K except that in the same pH range the plateau has not been reached.

Competition experiments with other anions present in biological fluids for which the interaction with Eu-DO3A was already reported (e.g., carbonate and lactate)^24^ were performed at pH 7.0. The Z-spectra reported in Figure S2 clearly showed that the presence of bicarbonate and lactate did not affect the ^31^P CEST contrast. Measurements carried out by performing a titration of a solution containing 10 mM P_i_ and 40 μM Eu-DO3A with increasing concentration of lactate from 0 to 20 mM displayed that there is no ST difference up to 20 mM lactate, thus suggesting that the competition starts when the concentration of lactate is much higher than that of P_i_ (see the Supporting Information).

To assess the potential of this approach in imaging applications, ^31^P MRI-CEST images and localized spectroscopy experiments were carried out on a solution containing 40 μM Eu-DO3A and 10 mM P_i_ at pH 7.0 on a 7 T preclinical MRI scanner equipped with a ^1^H/^31^P double-resonant-volume radio-frequency coil (40 mm).

The parameters for ^31^P MRI-CEST image acquisition are the following: pulse sequence Turbo RARE spin–echo, TE 13.9 ms, TR 10 s, averages 384, rare factor 8, slice 1, slice thickness 20 mm, FOV 40 × 40 mm, matrix 32 × 32, and excitation bandwidth 35088 Hz. ST module: block pulse, length 2 s, bandwidth 0.6 Hz, amplitude 12 μT, and total acquisition time 4 h 16 min. The saturation pulse was placed off-resonance (+134 ppm offset) and on-resonance (−134 ppm offset) and referred to the frequency of P_i_^free^. The Z-spectrum and on- and off-resonance images (Δω ± 134 ppm) are reported in Figure 3. The difference in the intensity of the ^31^P signal in the MRI configuration resulted in a CEST effect of 50%. Interestingly, because of the lowering of the magnetic field strength from 14 to 7 T, this result was obtained using a saturating pulse with significantly lower amplitude (12 μT vs 22 μT). The parameters used for ^31^P MRI-CEST localized spectroscopy are the following: pulse sequence image-selected in vivo spectroscopy (ISIS), TR 10 s, averages 2 (16 total ISIS average), saturation frequency range ±150 ppm, voxel size 40 × 40 × 20 mm, block pulse, amplitude 12 μT, acquisition time per single point 38 s; total acquisition time 3h 12 min.

**Figure 3:**
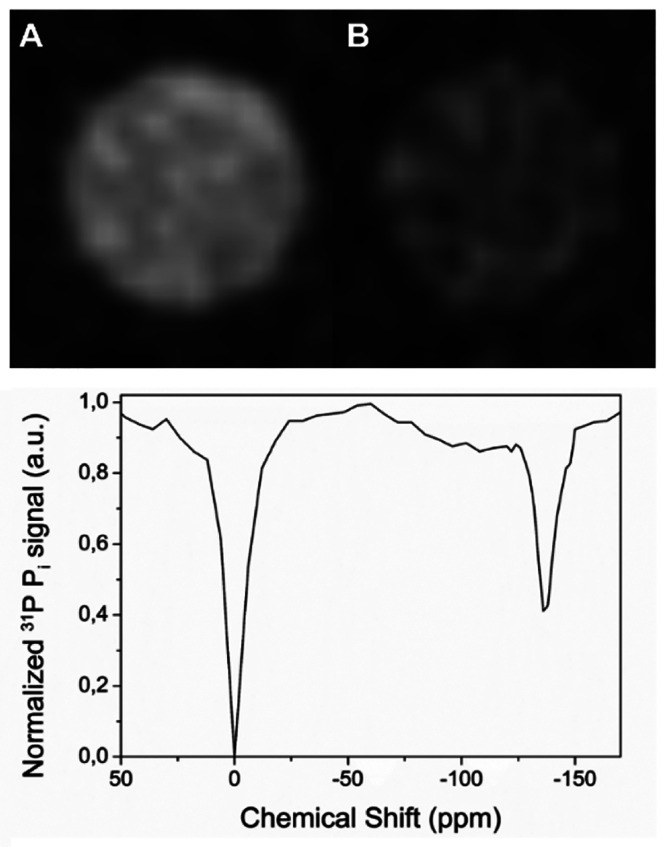
Top: Off-resonance (A) and on-resonance (B) ^31^P MRI-CEST of a solution of 40 μM Eu-DO3A and 10 mM P_i_ (pH 7.0) acquired at +134/–134 ppm at 7.0 T (ST % = 50). Bottom: ^31^P Z-spectrum of the same solution in the MRS-CEST configuration.

These results highlight the possibility of measuring a ^31^P ST % value by performing an on/off experiment in MRS-CEST localized spectroscopy in less than 2 min. The potential of the method for cellular imaging applications was tested by ex vivo labeling murine breast cancer cells (TS/A).^25^ The Eu-DO3A complex was entrapped in the cytosol by using the hypotonic swelling method, which consists of inducing a transient opening of pores on the cellular membrane when the cells are suspended in hypotonic solutions.^26^ During the hypotonic phase, Eu-DO3A quickly entered the cells, and after few minutes, the isotonicity was restored and the cells were washed to remove the noninternalized complex. The internalization efficiency was assessed by inductively coupled plasma mass spectrometry analyses, and an amount of Eu-DO3A of ca. 120 μM/cell was calculated. The total P_i_ concentration in the cell pellet was determined by NMR by integrating the P_i_ signal against a phosphocreatine standard on the cell lysate, and it was found to be 7.1 mM. Next, a pellet of about 2 × 10^7^ labeled cells was subjected to ^31^P MRI-CEST experiment. A ST % value of 20% was measured (Figure 4), thus demonstrating the feasibility of this approach for cellular MRI applications.

**Figure 4:**
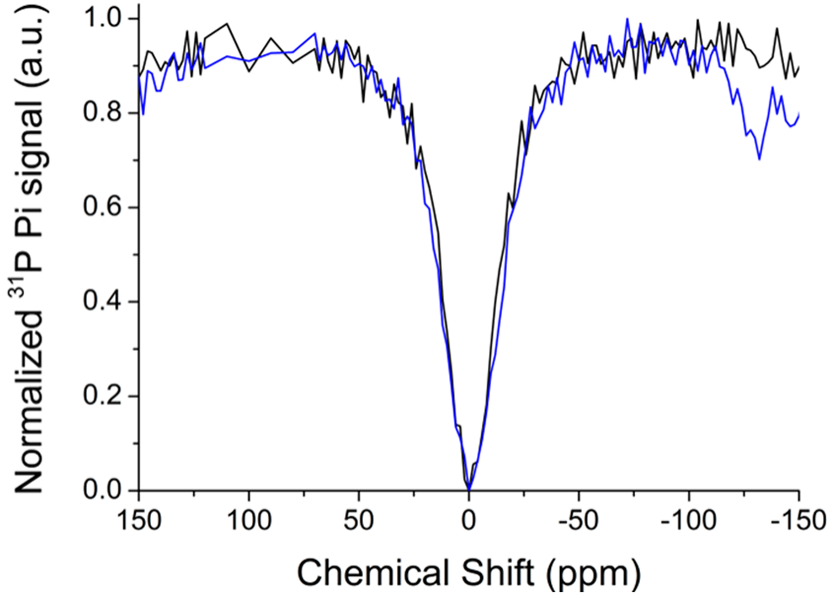
^31^P Z-spectrum of TS/A cells labeled with Eu-DO3A (blue line) and unlabeled (black line). The P_i_^bound^ peak appeared around −115 ppm.

Interestingly, the Δω value between P_i_^bound^ and P_i_^free^ in the Eu-DO3A-labeled cells was significantly lower than the value observed in aqueous solution at the same pH (assuming an intracellular pH around 7.2/7.4) and temperature (−115 ppm vs −134 ppm). The origin of the shift might arise, at least in part, from the nonisotropic cellular environment and from possible subtle changes in the molecular geometry of the ternary adduct in the intracellular environment.

In conclusion, the herein-reported results demonstrate that endogenous P_i_ in the presence of the Eu-DO3A complex can generate an efficient ^31^P CEST effect, with a detection sensitivity 3 orders of magnitude higher than the ^1^H detection of conventional paraCEST agents. However, the low concentration of the detected spins implies an overall decrease of the NMR detectability, thus requiring longer acquisition times. Nevertheless, the switch from MRI to MRS setting, where the CEST effect is measured in a selected volume, in front of the loss of spatial localization of the contrast, one may have the advantage of completing the experiment in a shorter time. Given the small lower limit of detection, we surmised that a possible application could be in the field of cellular MRI, where cells could be monitored without the need to load them with a high amount of labeling agent. In this proof-of-concept, we have shown that labeling cells with the Eu-DO3A complex at low micromolar concentration is sufficient for activation of the ^31^P CEST contrast associated with the intracellular P_i_. Several other applications can be envisaged. In the extracellular region, where an intravenously injected dose of Eu-DO3A distributes, the method may allow an accurate pH mapping. Inside the cell, the method may be applied to detect creatin kinase activity, for which ^1^H CEST detection, based on the signals of creatin and phosphocreatine, has been recently proposed.^27^

More work will allow improvement of the performance of this approach in terms of acquisition conditions and typology of the paramagnetic shift reagent. However, the herein-reported observations definitively show that a new chapter is now open in the field of multinuclear paraCEST domain with detection of the heteronuclear CEST response of endogenous molecules.

## Supporting Information Available

Sample preparation, cell labeling experiments, ^31^P CEST experiments for the NMR configuration, ^31^P CEST (on/off) experiments for the MRI configuration, ^31^P CEST (Z-spectrum) experiments for the MRS configuration, and Figures S1–S3 (PDF)
