# Supplementary material for: 31P ParaCEST: 31P MRI-CEST Imaging Based on the Formation of a Ternary Adduct between Inorganic Phosphate and Eu-DO3A
Source: Inorg Chem. 2022 Nov 29;61(49):19663–7. doi: 10.1021/acs.inorgchem.2c03329 (PMC9946289; doi:10.1021/acs.inorgchem.2c03329)
Supplement: Supplementary file 1 — ic2c03329_si_001.pdf [file ic2c03329_si_001.pdf]

Supporting Information

^{31}P-ParaCEST: ^{31}P MR-CEST Imaging based on the formation of a ternary adduct between inorganic phosphate and Eu-DO3A

Giulia Vassallo[a], Francesca Garello[a], Silvio Aime[b], Enzo Terreno[a] and Daniela Delli Castelli[a]*

[a] Department of Molecular Biotechnology and Health Science, University of Turin, Via Nizza 52, 10126 Turin, Italy

[b] IRCCS SDN SynLab, Via E.Gianturco 113, 80143 Napoli, Italy

Samples preparation

All chemicals were purchased from Sigma-Aldrich. Eu-DO3A complex was kindly provided by Bracco Imaging S.p.A. A stock solution of Eu-DO3A with a final concentration of 4 mM and a 10 mM solution of Pi (obtained dissolving KH_2PO_4 and then adjusting pH to 7). Dilutions of the paramagnetic complex to the desired concentration was carried out using Pi 10 mM. pH adjustments were done using 1 M NaOH or 1 M HCl.

Cell labeling experiments

TS/A cells derived from a spontaneous BALB/c mammary carcinoma were used. The cells were cultured in RPMI-1640 w/L-glutamine supplemented with 10% (*v*/v) fetal bovine serum (FBS) and 100 U/mL penicillin/streptomycin (P/S). Cells were grown at 37°C, with 5% CO_2.

TS/A cells were grown in a 175 cm^2 flask (Thermo-Fisher) at a density of ~20 x 10^6 per flask. Cells were detached by incubation with trypsin, resuspended in the medium, and centrifuged (1100 rpm / 21°C / 5 min) to eliminate the trypsin from the pellet. The cellular pellet, containing around 60 x 10^6 cells, was suspended in medium and aliquoted in 14 falcons (around 5 x 10^6 cells/falcon). First, 1 mL solution of PBS 160 mOsm pH 7.4 containing 2 mM EuDO3A was added to each falcon, followed by incubation for 30 min at 37°C, 5% CO_2. Then, 1 mL of hyperosmotic PBS 384 mOsm pH 7.4 was added and the falcons were again incubated for 15 min, to restore the isosmotic condition. At the end of the incubation time, two washing cycles with PBS pH 7.4 274 mOsm, followed by centrifugation of the sample, were performed to eliminate the excess of non-internalized EuDO3A. The third wash with HEPES/NaCl buffer pH 7.4 274 mOsm was performed to remove the phosphate residue from the extracellular environment. The cells were then suspended in HEPES/NaCl buffer and collected in a single 50 mL falcon tube to create a unique pellet, counting around 2 x 10^7 cells.

^{31}P CEST experiments: NMR configuration

^{31}P NMR spectra were acquired with a Bruker Avance 600 operating at 14 T. 5 mm NMR tubes were used. Tubes were filled with 400 µL of the solution of the paramagnetic complex, and a capillary filled with D_2O was used to allow the lock of the frequency. ^{31}P Z-spectra for each sample were acquired. Experimental conditions: frequency range: ± 170 ppm, T 298 K = 25°C, 32 scans, saturation pulse intensity 22 mT.

^{31}P CEST (on/off) experiments: MRI configuration

^{31}P CEST-MRI experiments were performed with a Bruker Pharmascan operating at 7 T and equipped with a ^{1}H/^{31}P double resonant volume RF coil (40 mm).

The following acquisition parameters were used: pulse sequence Turbo RARE spin-echo, TE 13.9 ms, TR 10 s, Averages 384, Rare Factor 8, Slice 1, Slice Thickness 20 mm, FOV 40 x 40 mm, Matrix 32 x 32, Excitation Bandwidth 35088 Hz. Saturation Transfer module: Block Pulse, Length 2 s, Bandwidth 0.6 Hz, Amplitude 12 µT, Total acquisition time = 4 h 16 min. Saturation pulse was placed off- (+134 ppm offset) and on- (-134 ppm offset) resonance referred to the frequency of Pi^{free}.

^{31}P CEST (Z-spectrum) experiments: MRS configuration

^{31}P CEST-MRS experiments were performed with Pharmascan operating at 7 T and equipped with a $^{1}H/^{31}P$ double resonant volume RF coil (40 mm).

The following acquisition parameters were used: pulse sequence Image-Selected In vivo Spectroscopy (ISIS), TR 10 s, Averages 2 (16 total ISIS average), saturation frequency range ± 150 ppm, voxel size 40 x 40 x 20 mm, Block pulse, Amplitude 12 µT, total acquisition time = 3h 12 min.

Figures

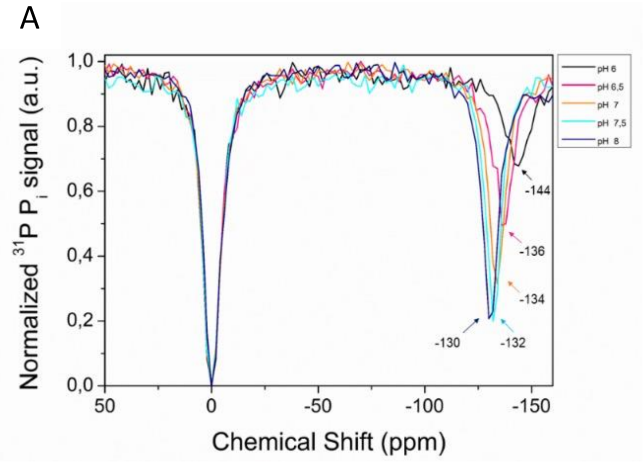

Fig. S1) Z-spectra at different pH in the pH range 6-8. At 295 K for a solution of 40 µM Eu-DO3A and 10 mM Pi

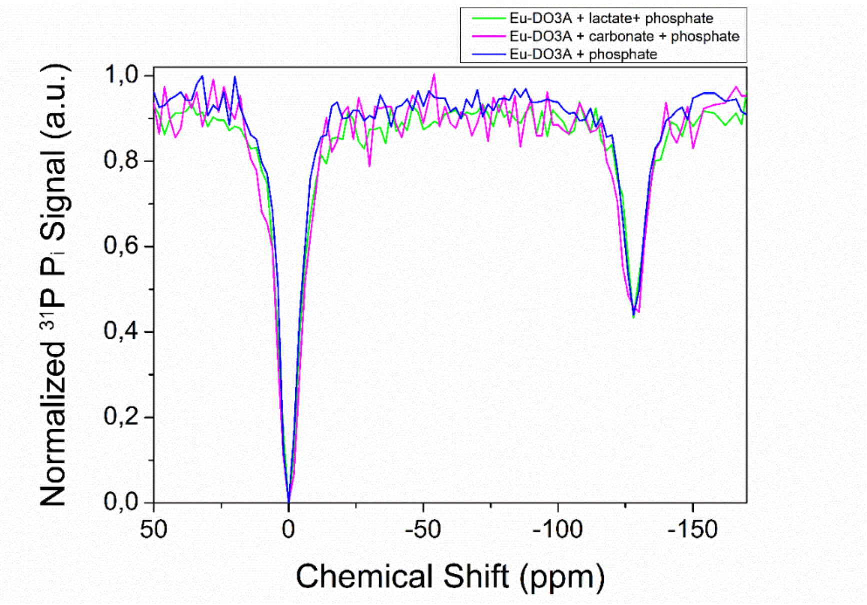

Fig. S2: ^{31}P Z-spectra overlay of three different solutions. In blue the control solution of 40 μM Eu-DO3A and 10 mM Pi is displayed. The same solution was added with 10 mM of bicarbonate (pink trace) or 5 mM of lactate (green).

A titration of a solution containg 10 mM Pi and 40μM EuDO3A with increasingly concentration of lactate from 0 to 20 mM with step of 5 mM, displayed that there is no Saturation transfer difference up to 20 mM of lactate, thus suggesting that the competition starts when the concentration of lactate is much higher than Pi.

Lactate concentration	ST%
0	48%
5mM	49%
10 mM	47%
20 mM	22%

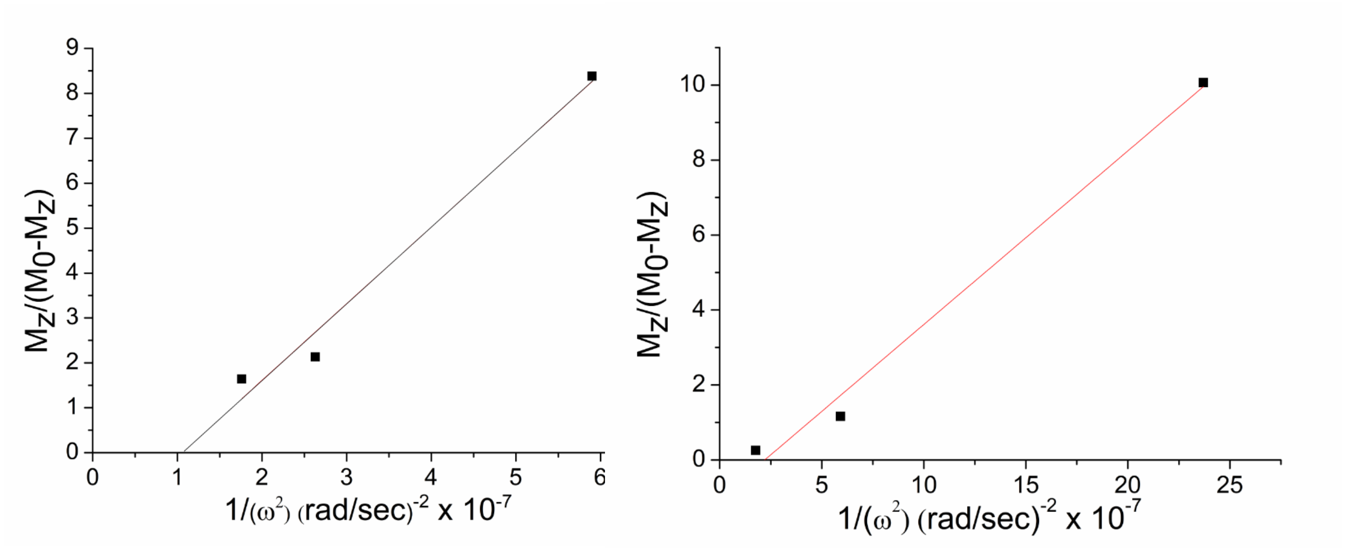

Fig. S3: Omega plot at 295 K left and 310 K right
